# μ-Adipato-bis­[chlorido(2,2′:6′,2′′-terpyridine)­copper(II)] tetra­hydrate

**DOI:** 10.1107/S1600536810027005

**Published:** 2010-07-14

**Authors:** Hong-Zhen Xie, Yan-Guang Zhang

**Affiliations:** a State Key Laboratory Base of Novel Functional Materials and Preparation Science, Faculty of Materials Science and Chemical Engineering, Ningbo University, Ningbo, Zhejiang 315211, People’s Republic of China

## Abstract

In the title compound, [Cu_2_(C_6_H_8_O_4_)Cl_2_(C_15_H_11_N_3_)_2_]·4H_2_O, the dinuclear copper complex is located on a crystallographic inversion centre. Each Cu atom is in a distorted square-pyramidal coordination environment, with one O atom of an adipate dianion and three N atoms from the 2,2′:6′,2′′-terpyridine ligand occupying the basal plane, and one chlorine in the apical site. In addition, there is weak Cu—O inter­action opposite of the chlorine with a distance of 2.768 (1) Å. The adipate ligand adopts a *gauche–anti–gauche* conformation. The inter­stitial water mol­ecules form hydrogen-bonded tertramers that are connected to the complexes *via* O—H⋯O and O—H⋯Cl hydrogen bonds, thus leading to the formation of tightly hydrogen-bonded layers extending perpendicular to the *b*-axis direction.

## Related literature

For general background to the use of saturated aliphatic dicarboxyl­ate ligands as flexible spacer ligands, see: Forster & Cheetham (2002 ▶); Vaidhyanathan *et al.* (2002 ▶); Zheng, Lin *et al.* (2008 ▶). For related structures, see: Zheng, Cheng *et al.* (2008 ▶). 
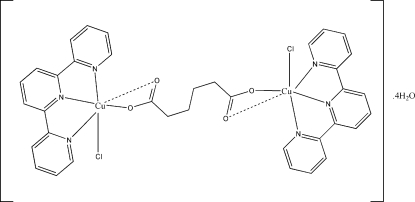


## Experimental

### 

#### Crystal data

[Cu_2_(C_6_H_8_O_4_)Cl_2_(C_15_H_11_N_3_)_2_]·4H_2_O
*M* *_r_* = 880.70
Triclinic, 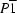

*a* = 8.2334 (16) Å
*b* = 9.5678 (19) Å
*c* = 11.548 (2) Å
α = 83.42 (3)°
β = 81.69 (3)°
γ = 84.38 (3)°
*V* = 891.2 (3) Å^3^
*Z* = 1
Mo *K*α radiation
μ = 1.41 mm^−1^
*T* = 298 K
0.25 × 0.22 × 0.07 mm

#### Data collection

Rigaku R-AXIS RAPID diffractometer
Absorption correction: multi-scan (*ABSCOR*; Higashi, 1995 ▶) *T*_min_ = 0.680, *T*_max_ = 0.892
8834 measured reflections
4046 independent reflections
3751 reflections with *I* > 2σ(*I*)
*R*_int_ = 0.016

#### Refinement

*R*[*F* ^2^ > 2σ(*F* ^2^)] = 0.025
*wR*(*F* ^2^) = 0.080
*S* = 1.25
4046 reflections
245 parameters
H-atom parameters constrained
Δρ_max_ = 0.48 e Å^−3^
Δρ_min_ = −0.51 e Å^−3^

### 

Data collection: *RAPID-AUTO* (Rigaku, 1998 ▶); cell refinement: *RAPID-AUTO*; data reduction: *CrystalStructure* (Rigaku/MSC, 2002 ▶); program(s) used to solve structure: *SHELXS97* (Sheldrick, 2008 ▶); program(s) used to refine structure: *SHELXL97* (Sheldrick, 2008 ▶); molecular graphics: *ORTEPII* (Johnson, 1976 ▶); software used to prepare material for publication: *SHELXL97*.

## Supplementary Material

Crystal structure: contains datablocks global, I. DOI: 10.1107/S1600536810027005/zl2287sup1.cif

Structure factors: contains datablocks I. DOI: 10.1107/S1600536810027005/zl2287Isup2.hkl

Additional supplementary materials:  crystallographic information; 3D view; checkCIF report

## Figures and Tables

**Table 1:** Hydrogen-bond geometry (Å, °)

*D*—H⋯*A*	*D*—H	H⋯*A*	*D*⋯*A*	*D*—H⋯*A*
O3—H3*C*⋯O4^i^	0.87	1.94	2.767 (2)	160
O3—H3*D*⋯O1	0.77	2.08	2.829 (2)	163
O4—H4*C*⋯Cl	0.93	2.32	3.194 (2)	156
O4—H4*D*⋯O3^ii^	0.88	2.02	2.805 (2)	148

Symmetry codes: (i) 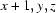; (ii) 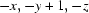.

**Fig. 1.**
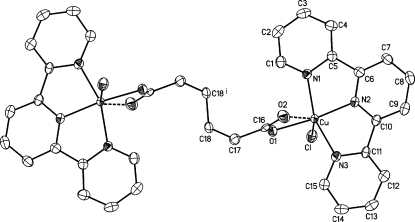
ORTEP view of complex molecule of the title compound. Displacement ellipsoids are drawn at the 45% probability level (i = -x + 1, -y + 1, -z + 1). H atoms and lattice water molecules are omitted for clarity.

**Fig. 2.**
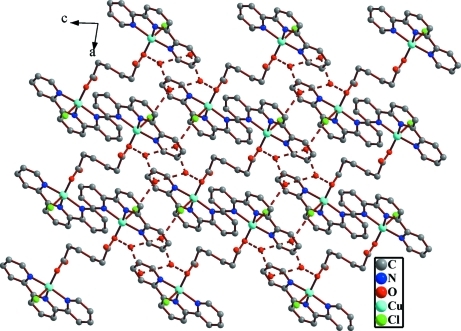
The hydrogen bonded layer perpendicular to the b-direction in the title compound. H atoms were omitted for clarity and dashed lines symbolize hydrogen bonds.

## supplementary crystallographic information

### Comment

Different from the more rigid dicarboxylate spacer ligands, saturated aliphatic dicarboxylate ligands are conformationally more flexible with a larger coordination versatility and as such they are viewed as important flexible spacer ligands (Forster & Cheetham, 2002; Vaidhyanathan *et al.*, 2002; Zheng, Lin *et al.*, 2008). Among these, the adipate dianion has often been used as a bridging ligand to construct dinuclear complexes (Zheng, Cheng *et al.*, 2008). In our recent research, we have been interested in the polydentate N-donor 2,2':6',2''-terpyridine which we have used together with bridging dicarboxylate ligands to construct polynuclear complexes. We report herein the synthesis and crystal structure of a new complex, [Cu_2_(C_15_H_11_N_3_)_2_(C_6_H_8_O_4_)Cl_2_].4H_2_O.

In the centrosymmetric dinuclear copper complex, two [Cu_2_(C_15_H_11_N_3_)_2_(C_6_H_8_O_4_)Cl_2_] moieties are bridged by an adipate ligand with a Cu···Cu separation in the dimer of 9.715 (2) Å (Fig. 1). The adipate ligand adopts a gauche-anti-gauche conformation. Each Cu atom is in a distorted square pyramidal coordination environment, with one O atom of an adipate dianion and three N atoms from the 2,2':6',2''-terpyridine ligand occupying the basal plane, and one chlorine in the apical site. In addition, there is a weak Cu-O interaction opposite of the chlorine with a distance of 2.768 (1) Å.

The interstitial water molecules are interacting with the metal complexes via hydrogen bonding interactions (Table 1). There are three kinds of hydrogen bonds: From one of the lattice water molecule to the coordinated oxygen atom of the carboxylate group, from the other water molecule towards a chlorine atom of one of the ligands, and between the water molecules themselves, which are arranged as tetramers in planar squares. In this way each of the water tetramers ties together four different complexes via H bonds to each two chlorines and two carboxylate oxygen atoms. The complexes in turn are hydrogen bonded to four of the water tetramers, thus leading to the formation of hydrogen bonded layers that extend perpendicular to the b-direction of the unit cell. (Fig.2).

### Experimental

Dropwise addition of 1 *M* aqueous Na_2_CO_3_ (3.0 ml) to a stirred aqueous solution of CuCl_2_.6H_2_O (0.1215 g, 0.50 mmol) in H_2_O (5.0 ml) produced a blue CuCO_3_ precipitate, which was centrifuged and washed with water until no Cl^-^ was detected in the supernatant. The resulting solid was added to a solution of adipic acid (0.0731, 0.50 mmol) and 2,2':6',2''-terpyridine (0.1166 g, 0.50 mmol) in 20 ml mixed solvent of H_2_O and CH_3_OH (v:v = 1:1). The mixture was stirred for half an hour and filtered, and the dark green filtrate (pH = 5.01) was left standing at room temperature. Green plate-like crystals were obtained several days later (Yield: ca. 23% based on Cu).

### Refinement

H atoms bonded to C atoms were placed in geometrically calculated positions and refined using a riding moldel with C–H = 0.93–0.97 Å and *U*_iso_(H) = 1.2 Ueq(C). H atoms of water were found in difference Fourier syntheses and fixed as initially found.

### Crystal data

**Table:**

[Cu_2_(C_6_H_8_O_4_)Cl_2_(C_15_H_11_N_3_)_2_]·4H_2_O	*Z* = 1
*M**_r_* = 880.70	*F*(000) = 452
Triclinic, *P*1	*D*_x_ = 1.641 Mg m^−^^3^
Hall symbol: -P 1	Mo *K*α radiation, λ = 0.71073 Å
*a* = 8.2334 (16) Å	Cell parameters from 7883 reflections
*b* = 9.5678 (19) Å	θ = 3.0–27.4°
*c* = 11.548 (2) Å	µ = 1.41 mm^−^^1^
α = 83.42 (3)°	*T* = 298 K
β = 81.69 (3)°	Plate, green
γ = 84.38 (3)°	0.25 × 0.22 × 0.07 mm
*V* = 891.2 (3) Å^3^	

### Data collection

**Table:**

Rigaku R-AXIS RAPID diffractometer	4046 independent reflections
Radiation source: fine-focus sealed tube	3751 reflections with *I* > 2σ(*I*)
graphite	*R*_int_ = 0.016
Detector resolution: 0 pixels mm^-1^	θ_max_ = 27.5°, θ_min_ = 3.2°
ω scans	*h* = −10→10
Absorption correction: multi-scan (*ABSCOR*; Higashi, 1995)	*k* = −12→12
*T*_min_ = 0.680, *T*_max_ = 0.892	*l* = −14→14
8834 measured reflections	

### Refinement

**Table:**

Refinement on *F*^2^	Primary atom site location: structure-invariant direct methods
Least-squares matrix: full	Secondary atom site location: difference Fourier map
*R*[*F*^2^ > 2σ(*F*^2^)] = 0.025	Hydrogen site location: inferred from neighbouring sites
*wR*(*F*^2^) = 0.080	H-atom parameters constrained
*S* = 1.25	*w* = 1/[σ^2^(*F*_o_^2^) + (0.0414*P*)^2^ + 0.4176*P*] where *P* = (*F*_o_^2^ + 2*F*_c_^2^)/3
4046 reflections	(Δ/σ)_max_ = 0.001
245 parameters	Δρ_max_ = 0.48 e Å^−^^3^
0 restraints	Δρ_min_ = −0.51 e Å^−^^3^

### Special details

**Table:**

Geometry. All e.s.d.'s (except the e.s.d. in the dihedral angle between two l.s. planes) are estimated using the full covariance matrix. The cell e.s.d.'s are taken into account individually in the estimation of e.s.d.'s in distances, angles and torsion angles; correlations between e.s.d.'s in cell parameters are only used when they are defined by crystal symmetry. An approximate (isotropic) treatment of cell e.s.d.'s is used for estimating e.s.d.'s involving l.s. planes.
Refinement. Refinement of *F*^2^ against ALL reflections. The weighted *R*-factor *wR* and goodness of fit *S* are based on *F*^2^, conventional *R*-factors *R* are based on *F*, with *F* set to zero for negative *F*^2^. The threshold expression of *F*^2^ > σ(*F*^2^) is used only for calculating *R*-factors(gt) *etc*. and is not relevant to the choice of reflections for refinement. *R*-factors based on *F*^2^ are statistically about twice as large as those based on *F*, and *R*- factors based on ALL data will be even larger.

### Fractional atomic coordinates and isotropic or equivalent isotropic displacement parameters (Å^2^)

**Table:**

	*x*	*y*	*z*	*U*_iso_*/*U*_eq_	
Cu	0.13815 (3)	0.79530 (2)	0.258347 (18)	0.00798 (8)	
Cl	0.00080 (5)	0.61527 (4)	0.17386 (4)	0.01293 (10)	
O1	0.31683 (16)	0.65682 (13)	0.29993 (11)	0.0110 (3)	
O2	0.41422 (18)	0.84454 (14)	0.35396 (12)	0.0158 (3)	
O3	0.40877 (18)	0.42538 (14)	0.16550 (12)	0.0173 (3)	
H3C	0.5094	0.4458	0.1431	0.021*	
H3D	0.3784	0.4760	0.2132	0.021*	
O4	−0.30631 (19)	0.54262 (18)	0.05804 (13)	0.0244 (3)	
H4C	−0.2000	0.5478	0.0749	0.029*	
H4D	−0.2994	0.5637	−0.0183	0.029*	
N1	−0.00477 (19)	0.78588 (16)	0.41703 (13)	0.0093 (3)	
N2	0.01050 (18)	0.97845 (15)	0.24509 (13)	0.0089 (3)	
N3	0.24603 (19)	0.88021 (16)	0.10055 (13)	0.0097 (3)	
C1	−0.0037 (2)	0.67745 (19)	0.50071 (16)	0.0121 (3)	
H1A	0.0753	0.6020	0.4902	0.014*	
C2	−0.1158 (2)	0.6730 (2)	0.60277 (16)	0.0139 (4)	
H2A	−0.1120	0.5962	0.6598	0.017*	
C3	−0.2338 (2)	0.7858 (2)	0.61759 (16)	0.0151 (4)	
H3A	−0.3115	0.7851	0.6846	0.018*	
C4	−0.2350 (2)	0.90009 (19)	0.53138 (16)	0.0131 (4)	
H4A	−0.3123	0.9771	0.5404	0.016*	
C5	−0.1188 (2)	0.89694 (18)	0.43166 (16)	0.0098 (3)	
C6	−0.1046 (2)	1.01126 (19)	0.33414 (16)	0.0104 (3)	
C7	−0.1938 (2)	1.14257 (19)	0.33008 (16)	0.0128 (4)	
H7A	−0.2752	1.1655	0.3912	0.015*	
C8	−0.1577 (2)	1.23842 (19)	0.23184 (17)	0.0138 (4)	
H8A	−0.2157	1.3269	0.2272	0.017*	
C9	−0.0360 (2)	1.20397 (19)	0.14023 (16)	0.0126 (4)	
H9A	−0.0112	1.2683	0.0748	0.015*	
C10	0.0474 (2)	1.06991 (18)	0.14981 (16)	0.0100 (3)	
C11	0.1807 (2)	1.01092 (18)	0.06390 (15)	0.0093 (3)	
C12	0.2341 (2)	1.07967 (19)	−0.04513 (16)	0.0122 (3)	
H12A	0.1876	1.1691	−0.0687	0.015*	
C13	0.3592 (2)	1.0118 (2)	−0.11900 (16)	0.0134 (4)	
H13A	0.3983	1.0557	−0.1924	0.016*	
C14	0.4240 (2)	0.8782 (2)	−0.08133 (16)	0.0137 (4)	
H14A	0.5065	0.8308	−0.1296	0.016*	
C15	0.3649 (2)	0.81548 (19)	0.02906 (16)	0.0117 (3)	
H15A	0.4092	0.7258	0.0541	0.014*	
C16	0.4287 (2)	0.71750 (18)	0.33897 (15)	0.0095 (3)	
C17	0.5825 (2)	0.62512 (19)	0.36324 (16)	0.0121 (3)	
H17A	0.6498	0.6774	0.4025	0.015*	
H17B	0.6456	0.6031	0.2890	0.015*	
C18	0.5460 (2)	0.48718 (19)	0.43937 (15)	0.0117 (4)	
H18A	0.4804	0.4338	0.3997	0.014*	
H18C	0.6489	0.4311	0.4487	0.014*	

### Atomic displacement parameters (Å^2^)

**Table:**

	*U*^11^	*U*^22^	*U*^33^	*U*^12^	*U*^13^	*U*^23^
Cu	0.00828 (12)	0.00753 (11)	0.00710 (12)	0.00144 (7)	−0.00039 (8)	0.00098 (8)
Cl	0.0137 (2)	0.0122 (2)	0.0137 (2)	−0.00219 (15)	−0.00496 (16)	0.00018 (16)
O1	0.0104 (6)	0.0105 (6)	0.0121 (6)	0.0011 (5)	−0.0030 (5)	−0.0010 (5)
O2	0.0188 (7)	0.0106 (6)	0.0179 (7)	0.0001 (5)	−0.0016 (5)	−0.0031 (5)
O3	0.0191 (7)	0.0159 (6)	0.0161 (7)	−0.0004 (5)	0.0020 (5)	−0.0052 (5)
O4	0.0184 (8)	0.0409 (9)	0.0157 (7)	−0.0097 (7)	−0.0021 (6)	−0.0040 (7)
N1	0.0105 (7)	0.0089 (7)	0.0086 (7)	−0.0010 (5)	−0.0017 (5)	−0.0008 (6)
N2	0.0092 (7)	0.0083 (7)	0.0092 (7)	−0.0006 (5)	−0.0026 (6)	0.0001 (6)
N3	0.0098 (7)	0.0105 (7)	0.0090 (7)	−0.0011 (5)	−0.0020 (5)	−0.0001 (6)
C1	0.0117 (8)	0.0115 (8)	0.0133 (8)	−0.0008 (6)	−0.0034 (7)	−0.0004 (7)
C2	0.0171 (9)	0.0145 (8)	0.0108 (8)	−0.0047 (7)	−0.0037 (7)	0.0015 (7)
C3	0.0174 (9)	0.0176 (9)	0.0100 (8)	−0.0050 (7)	0.0025 (7)	−0.0024 (7)
C4	0.0139 (9)	0.0123 (8)	0.0129 (8)	−0.0017 (7)	0.0008 (7)	−0.0028 (7)
C5	0.0109 (8)	0.0086 (8)	0.0106 (8)	−0.0011 (6)	−0.0027 (6)	−0.0019 (7)
C6	0.0099 (8)	0.0116 (8)	0.0105 (8)	−0.0010 (6)	−0.0026 (6)	−0.0031 (7)
C7	0.0117 (9)	0.0128 (8)	0.0142 (9)	0.0018 (7)	−0.0026 (7)	−0.0045 (7)
C8	0.0147 (9)	0.0093 (8)	0.0177 (9)	0.0020 (6)	−0.0055 (7)	−0.0016 (7)
C9	0.0152 (9)	0.0102 (8)	0.0130 (8)	−0.0022 (7)	−0.0048 (7)	0.0017 (7)
C10	0.0102 (8)	0.0104 (8)	0.0100 (8)	−0.0017 (6)	−0.0035 (6)	0.0003 (7)
C11	0.0093 (8)	0.0089 (8)	0.0102 (8)	−0.0020 (6)	−0.0038 (6)	0.0011 (7)
C12	0.0133 (9)	0.0125 (8)	0.0116 (8)	−0.0034 (7)	−0.0035 (7)	−0.0002 (7)
C13	0.0140 (9)	0.0177 (9)	0.0092 (8)	−0.0068 (7)	−0.0013 (7)	−0.0003 (7)
C14	0.0117 (9)	0.0182 (9)	0.0114 (8)	−0.0030 (7)	0.0013 (7)	−0.0049 (7)
C15	0.0121 (9)	0.0109 (8)	0.0117 (8)	−0.0004 (6)	−0.0021 (7)	−0.0002 (7)
C16	0.0120 (8)	0.0100 (8)	0.0053 (7)	−0.0016 (6)	0.0016 (6)	0.0018 (6)
C17	0.0100 (8)	0.0144 (8)	0.0116 (8)	−0.0003 (6)	−0.0020 (6)	−0.0002 (7)
C18	0.0119 (8)	0.0122 (8)	0.0109 (8)	0.0036 (6)	−0.0044 (7)	−0.0012 (7)

### Geometric parameters (Å, °)

**Table:**

Cu—N2	1.9552 (16)	C5—C6	1.478 (3)
Cu—O1	1.9562 (14)	C6—C7	1.391 (2)
Cu—N3	2.0257 (17)	C7—C8	1.391 (3)
Cu—N1	2.0274 (16)	C7—H7A	0.9300
Cu—Cl	2.5067 (8)	C8—C9	1.393 (3)
O1—C16	1.294 (2)	C8—H8A	0.9300
O2—C16	1.239 (2)	C9—C10	1.395 (2)
O3—H3C	0.8658	C9—H9A	0.9300
O3—H3D	0.7734	C10—C11	1.483 (2)
O4—H4C	0.9308	C11—C12	1.385 (3)
O4—H4D	0.8764	C12—C13	1.397 (3)
N1—C1	1.335 (2)	C12—H12A	0.9300
N1—C5	1.356 (2)	C13—C14	1.383 (3)
N2—C6	1.337 (2)	C13—H13A	0.9300
N2—C10	1.343 (2)	C14—C15	1.387 (3)
N3—C15	1.337 (2)	C14—H14A	0.9300
N3—C11	1.358 (2)	C15—H15A	0.9300
C1—C2	1.387 (3)	C16—C17	1.513 (3)
C1—H1A	0.9300	C17—C18	1.530 (3)
C2—C3	1.389 (3)	C17—H17A	0.9700
C2—H2A	0.9300	C17—H17B	0.9700
C3—C4	1.392 (3)	C18—C18^i^	1.527 (3)
C3—H3A	0.9300	C18—H18A	0.9700
C4—C5	1.388 (3)	C18—H18C	0.9700
C4—H4A	0.9300		
			
N2—Cu—O1	157.68 (6)	C8—C7—H7A	121.0
N2—Cu—N3	79.61 (7)	C6—C7—H7A	121.0
O1—Cu—N3	99.40 (6)	C7—C8—C9	121.01 (17)
N2—Cu—N1	79.49 (7)	C7—C8—H8A	119.5
O1—Cu—N1	98.23 (6)	C9—C8—H8A	119.5
N3—Cu—N1	158.62 (6)	C8—C9—C10	117.87 (17)
N2—Cu—Cl	110.17 (5)	C8—C9—H9A	121.1
O1—Cu—Cl	92.15 (4)	C10—C9—H9A	121.1
N3—Cu—Cl	94.79 (5)	N2—C10—C9	120.32 (17)
N1—Cu—Cl	96.54 (5)	N2—C10—C11	112.50 (15)
C16—O1—Cu	110.59 (11)	C9—C10—C11	127.18 (17)
H3C—O3—H3D	102.7	N3—C11—C12	122.00 (16)
H4C—O4—H4D	104.5	N3—C11—C10	113.92 (15)
C1—N1—C5	119.25 (16)	C12—C11—C10	124.06 (16)
C1—N1—Cu	125.82 (13)	C11—C12—C13	118.57 (17)
C5—N1—Cu	114.70 (12)	C11—C12—H12A	120.7
C6—N2—C10	122.21 (15)	C13—C12—H12A	120.7
C6—N2—Cu	118.81 (12)	C14—C13—C12	119.01 (17)
C10—N2—Cu	118.88 (12)	C14—C13—H13A	120.5
C15—N3—C11	119.08 (16)	C12—C13—H13A	120.5
C15—N3—Cu	125.77 (12)	C13—C14—C15	119.40 (17)
C11—N3—Cu	114.98 (12)	C13—C14—H14A	120.3
N1—C1—C2	122.57 (17)	C15—C14—H14A	120.3
N1—C1—H1A	118.7	N3—C15—C14	121.92 (17)
C2—C1—H1A	118.7	N3—C15—H15A	119.0
C1—C2—C3	118.37 (18)	C14—C15—H15A	119.0
C1—C2—H2A	120.8	O2—C16—O1	122.70 (17)
C3—C2—H2A	120.8	O2—C16—C17	121.07 (17)
C2—C3—C4	119.53 (18)	O1—C16—C17	116.22 (15)
C2—C3—H3A	120.2	C16—C17—C18	113.19 (15)
C4—C3—H3A	120.2	C16—C17—H17A	108.9
C5—C4—C3	118.75 (17)	C18—C17—H17A	108.9
C5—C4—H4A	120.6	C16—C17—H17B	108.9
C3—C4—H4A	120.6	C18—C17—H17B	108.9
N1—C5—C4	121.52 (17)	H17A—C17—H17B	107.8
N1—C5—C6	113.96 (16)	C18^i^—C18—C17	112.17 (18)
C4—C5—C6	124.51 (16)	C18^i^—C18—H18A	109.2
N2—C6—C7	120.59 (17)	C17—C18—H18A	109.2
N2—C6—C5	112.72 (15)	C18^i^—C18—H18C	109.2
C7—C6—C5	126.68 (17)	C17—C18—H18C	109.2
C8—C7—C6	117.98 (17)	H18A—C18—H18C	107.9

Symmetry codes: (i) −*x*+1, −*y*+1, −*z*+1.

### Hydrogen-bond geometry (Å, °)

**Table:**

*D*—H···*A*	*D*—H	H···*A*	*D*···*A*	*D*—H···*A*
O3—H3C···O4^ii^	0.87	1.94	2.767 (2)	160
O3—H3D···O1	0.77	2.08	2.829 (2)	163
O4—H4C···Cl	0.93	2.32	3.194 (2)	156
O4—H4D···O3^iii^	0.88	2.02	2.805 (2)	148

Symmetry codes: (ii) *x*+1, *y*, *z*; (iii) −*x*, −*y*+1, −*z*.
